# Supplementary material for: Highly Sensitive and Specific Detection of Staphylococcal Enterotoxins SEA, SEG, SEH, and SEI by Immunoassay
Source: Toxins (Basel). 2021 Feb 9;13(2):130. doi: 10.3390/toxins13020130 (PMC7916246; doi:10.3390/toxins13020130)
Supplement: Supplementary file 1 [file toxins-13-00130-s001.pdf]

Supplementary Materials: Highly Sensitive and Specific Detection of Staphylococcal Enterotoxins SEA, SEG, SEH and SEI by Immunoassay

Cécile Féraudet Tarisse, Céline Goulard-Huet, Yacine Nia, Karine Devilliers, Dominique Marcé, Chloé Dambrune, Donatien Lefebvre, Jacques-Antoine Hennekinne and Stéphanie Simon

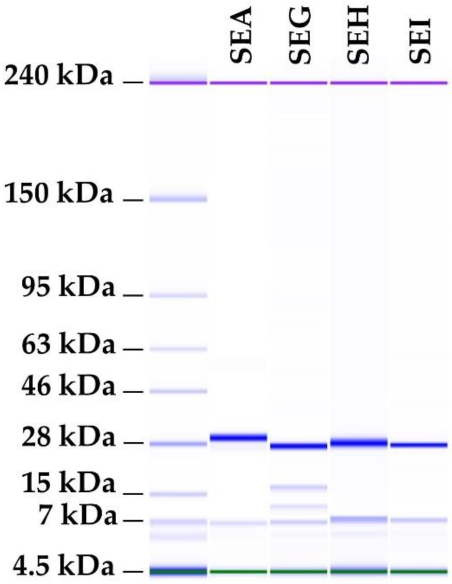

Figure S1. Microfluidic capillary gel electrophoresis for purified recombinant staphylococcal enterotoxins type A (SEA), G (SEG), H (SEH) and I (SEI) in reducing conditions (1 mg/mL) using the Protein 230 kit on the 2100 Bioanalyzer System (Agilent Technologies Inc., Santa Clara, CA, USA).

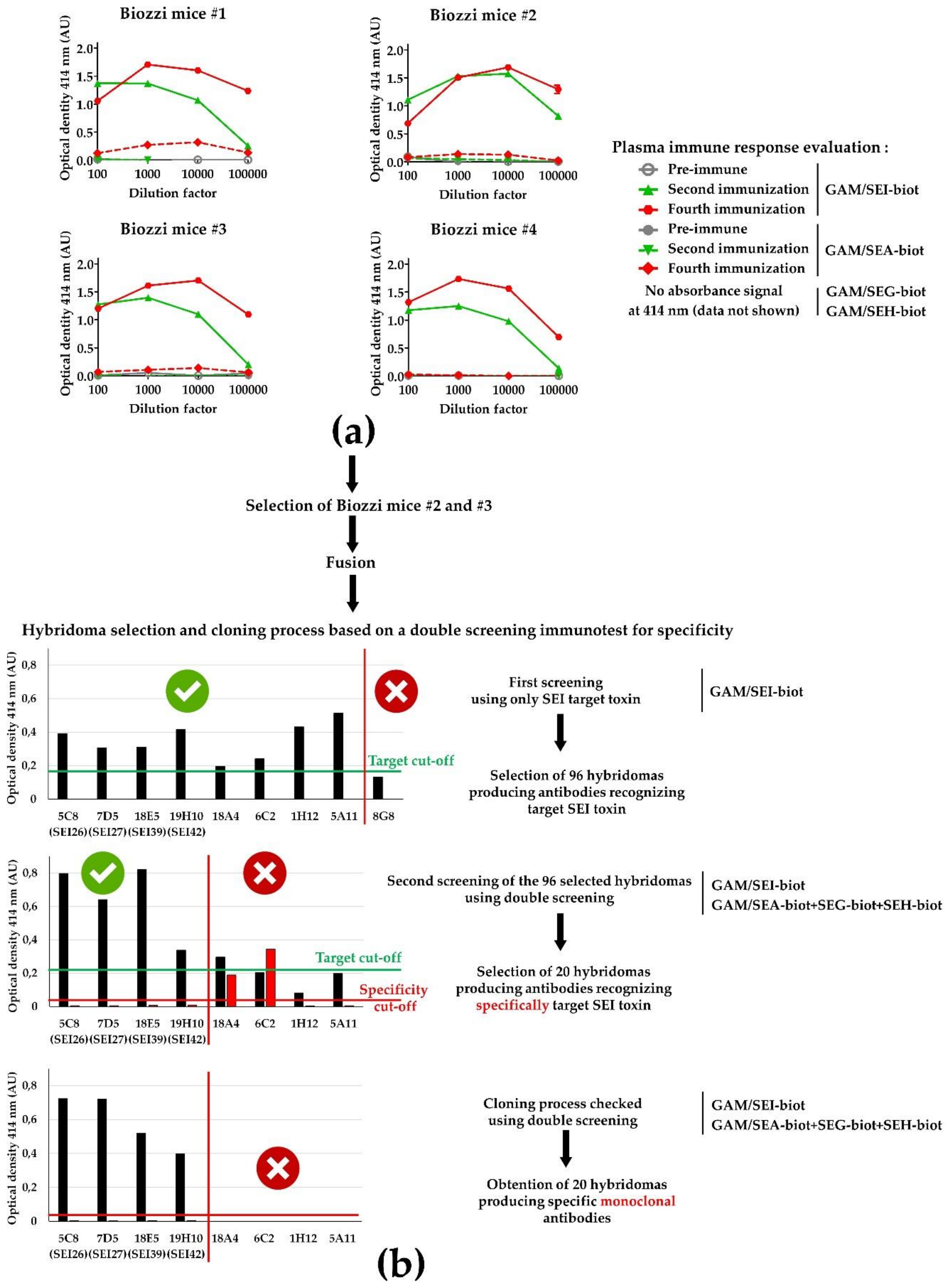

Figure S2. Process followed to select mAbs specific for their target toxin: illustration for SEI toxin. (**a**) Plasma immune response of the four Biozzi mice immunized 4 times at 3-week intervals with lab-made recombinant SEI toxin. Mice were bled before the first immunization (pre-immune) and two weeks after each immunization. Immune response evaluation was performed by ELISA using coated

goat anti-mouse Ig(G+M) antibodies (GAM) and biotinylated toxins combined with the use of acetylcholinesterase-labeled streptavidin conjugate for colorimetric analysis (see methods). *Biozzi* mice #2 and #3 were selected as they combine both low cross-reactivities towards SEA, SEG and SEH, and high anti-SEI titers (elevated signals for highly diluted plasma). (**b**) Evaluation of polyclonal and monoclonal response in hybridoma supernatants throughout the hybridoma selection and cloning process. Except for the first screening, antibody response was measured by two ELISAs (double screening), one for target sensitivity (coated GAM/biotinylated SEI), the second for specificity (coated GAM/ mixture of biotinylated recombinant SEA, SEG and SEH) (see methods). AU, Absorbance Unit.

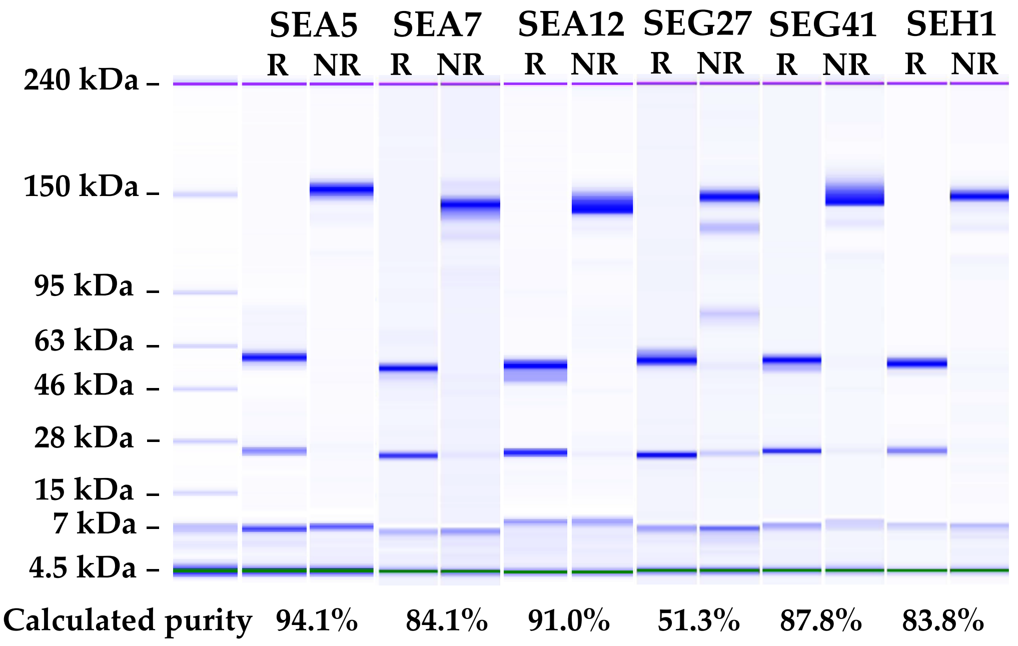

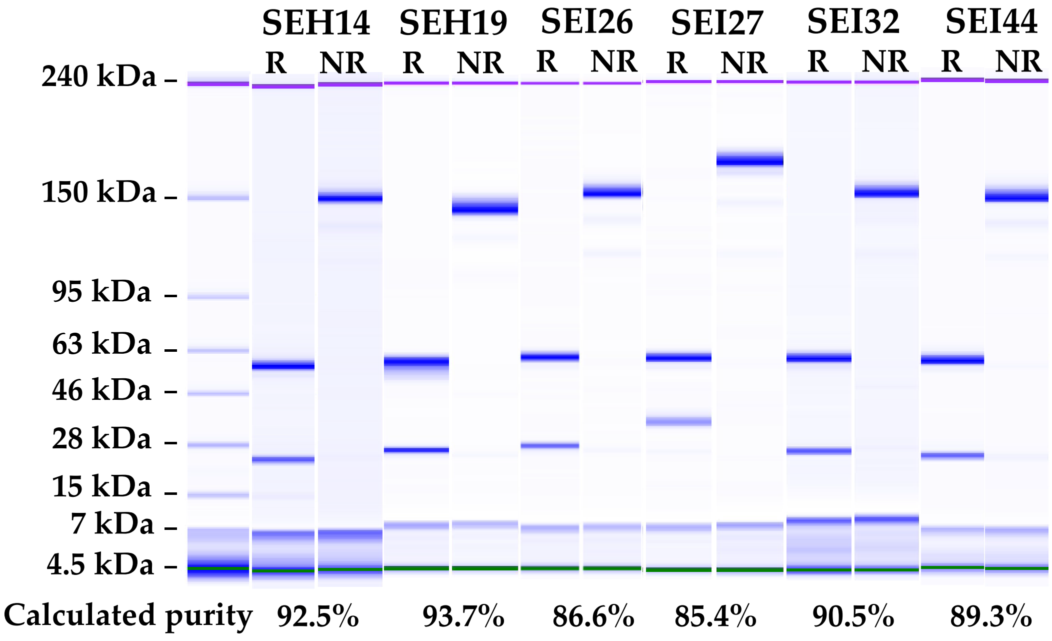

Figure S3. Microfluidic capillary gel electrophoresis for purified monoclonal antibodies (mAb) SEA5, SEA7, SEA12, SEG27, SEG41, SEH1, SEH14, SEH19, SEI26, SEI27, SEI32 and SEI44 in reducing (R) and non-reducing (NR) conditions (with mAb concentration between 1 mg/ml and 3 mg/mL) using the Protein 230 kit on the 2100 Bioanalyzer System (Agilent Technologies Inc., Santa Clara, CA, USA). Antibody purity was calculated from the ratio of 150 kDa peak area (i.e. intensity) to total peaks area in non-reducing conditions.

Table S1. List of the 79 monoclonal antibodies produced. Antibodies targeting possible similar, overlapping or nearby epitopes are indicated in the same line (i.e. unable to bind simultaneously to the target toxin). c, best capture mAbs. t, best tracer mAbs.

Immunogen	mAb	Isotype
**Recombinant lab-made SEA**	SEA5 (c,t), SEA12 (c,t)	IgG1
	SEA7 (c,t), SEA11 (c)	IgG1 (SEA7), IgG2b (SEA11)
	SEA1	IgG1
	SEA2	IgM
	SEA3	IgG2b
	SEA4	IgG1
	SEA13	IgM
	SEA14	IgM
	SEA15	IgG1
**Recombinant lab-made SEG**	SEG27 (t), SEG28 (t), SEG40	IgG2b (SEG27 and SEG28), IgG1 (SEG40)
	SEG22, SEG30 (c), SEG31 (c)	IgG1
	SEG25 (c), SEG39 (c,t)	IgG1
	SEG29, SEG32 (c), SEG33 (c)	IgG1
	SEG21 (c), SEG34 (c,t)	IgG1
	SEG35 (c), SEG41 (c,t)	IgG1
	SEG20	IgG1
	SEG24 (t)	IgG2a or IgG2b
	SEG26 (c, t)	IgG2a or IgG2c
	SEG37 (c, t)	IgG1
	SEG38 (c, t)	IgG1
**Recombinant lab-made SEH**	SEH2, SEH6 (c), SEH7, SEH8 (c), SEH11 (c), SEH12, SEH13, SEH14 (c,t), SEH15 (c), SEH17, SEH18	IgG1
	SEH4 (c), SEH5	IgG1
	SEH19 (c,t), SEH20 (c), SEH21	IgG1
	SEH1 (c,t)	IgG1
	SEH16 (c)	IgG1
**Recombinant lab-made SEI**	SEI22 (c,t), SEI23 (c), SEI28 (c), SEI31, SEI33 (c), SEI37 (c), SEI41 (c), SEI43	IgG1
	SEI24, SEI25, SEI26 (t), SEI32 (t), SEI34, SEI35, SEI40	IgG1

SEI21, SEI27 (c), SEI29, SEI36 (c,t), SEI39 (c), SEI44 (c,t)	IgG1
SEI1	IgG1
SEI2	IgG1
SEI3	IgG1
SEI4	IgG1
SEI5	IgG1
SEI6	IgG1
SEI7	IgG1
SEI30	IgG1
SEI38 (c,t)	IgG1

Table S2. Kinetic parameters of monoclonal antibodies (mAbs) for their target staphylococcal enterotoxin (SE). Kinetic parameters of the mAbs were determined by bio-layer interferometry in multi-cycle kinetics using lab-made recombinant target toxins and commercial SEA toxin as antigen. Mean and standard deviation values of equilibrium dissociation constant (K_D), association rate (k_{on}) and dissociation rate (k_{off}) were extracted from the curve fitting analysis of mAb association and dissociation to 3 to 6 target toxin successive dilutions (out of 7) and from 1 to 3 independent experiment(s). N.D.: not determined.

Target to × in	mAb	K_D (M)	k_{on} ($M^{-1}.s^{-1}$)	k_{off} (s^{-1})
**Recombinant lab-made SEA**	SEA5	$1.2 \times 10^{-10} \pm <1.0 \times 10^{-12}$	$6.8 \times 10^{5} \pm 6.9 \times 10^{2}$	$8.3 \times 10^{-5} \pm 3.5 \times 10^{-7}$
	SEA7	$3.6 \times 10^{-11} \pm <1.0 \times 10^{-12}$	$1.0 \times 10^{6} \pm 3.3 \times 10^{5}$	$3.7 \times 10^{-5} \pm 1.0 \times 10^{-5}$
	SEA11	$2.2 \times 10^{-10} \pm 8.0 \times 10^{-12}$	$9.6 \times 10^{4} \pm 5.9 \times 10^{2}$	$2.1 \times 10^{-5} \pm 7.6 \times 10^{-7}$
	SEA12	$5.6 \times 10^{-11} \pm <1.0 \times 10^{-12}$	$1.3 \times 10^{6} \pm 3.7 \times 10^{5}$	$7.6 \times 10^{-5} \pm 9.9 \times 10^{-6}$
**Commercial SEA**	SEA5	$5.2 \times 10^{-11} \pm <1.0 \times 10^{-12}$	$3.6 \times 10^{6} \pm 2.8 \times 10^{5}$	$1.9 \times 10^{-4} \pm 2.3 \times 10^{-5}$
	SEA7	$9.0 \times 10^{-12} \pm <1.0 \times 10^{-12}$	$3.6 \times 10^{6} \pm 6.7 \times 10^{5}$	$3.2 \times 10^{-5} \pm 1.2 \times 10^{-5}$
	SEA11	N.D.	N.D.	N.D.
	SEA12	$2.4 \times 10^{-11} \pm <1.0 \times 10^{-12}$	$1.8 \times 10^{7} \pm 7.0 \times 10^{5}$	$4.3 \times 10^{-4} \pm 4.3 \times 10^{-5}$
**Recombinant lab-made SEG**	SEG21	$4.1 \times 10^{-9} \pm 1.1 \times 10^{-11}$	$3.6 \times 10^{5} \pm 9.4 \times 10^{2}$	$1.4 \times 10^{-3} \pm 1.3 \times 10^{-6}$
	SEG24	$2.5 \times 10^{-10} \pm <1.0 \times 10^{-12}$	$5.5 \times 10^{5} \pm 6.1 \times 10^{2}$	$1.4 \times 10^{-4} \pm 3.8 \times 10^{-7}$
	SEG26	$2.0 \times 10^{-10} \pm <1.0 \times 10^{-12}$	$6.6 \times 10^{5} \pm 9.7 \times 10^{2}$	$1.3 \times 10^{-4} \pm 5.0 \times 10^{-7}$
	SEG27	$4.8 \times 10^{-11} \pm <1.0 \times 10^{-12}$	$1.8 \times 10^{6} \pm 4.2 \times 10^{3}$	$8.7 \times 10^{-5} \pm 5.5 \times 10^{-7}$
	SEG28	$9.0 \times 10^{-11} \pm 1.5 \times 10^{-12}$	$8.8 \times 10^{5} \pm 2.5 \times 10^{3}$	$7.9 \times 10^{-5} \pm 9.6 \times 10^{-7}$
	SEG30	$2.6 \times 10^{-10} \pm 1.5 \times 10^{-12}$	$4.0 \times 10^{5} \pm 6.8 \times 10^{2}$	$1.0 \times 10^{-4} \pm 5.9 \times 10^{-7}$
	SEG31	$3.2 \times 10^{-10} \pm 2.6 \times 10^{-12}$	$4.1 \times 10^{5} \pm 1.2 \times 10^{3}$	$1.3 \times 10^{-4} \pm 9.9 \times 10^{-7}$
	SEG32	$2.4 \times 10^{-10} \pm 1.0 \times 10^{-12}$	$6.8 \times 10^{5} \pm 1.3 \times 10^{3}$	$1.6 \times 10^{-4} \pm 6.2 \times 10^{-7}$
	SEG34	$3.6 \times 10^{-10} \pm 1.4 \times 10^{-12}$	$3.7 \times 10^{5} \pm 5.7 \times 10^{2}$	$1.3 \times 10^{-4} \pm 4.9 \times 10^{-7}$
	SEG37	$3.1 \times 10^{-10} \pm <1.0 \times 10^{-12}$	$8.2 \times 10^{5} \pm 1.2 \times 10^{3}$	$2.5 \times 10^{-4} \pm 5.0 \times 10^{-7}$
	SEG38	$1.3 \times 10^{-10} \pm <1.0 \times 10^{-12}$	$1.3 \times 10^{6} \pm 3.0 \times 10^{3}$	$1.6 \times 10^{-4} \pm 8.6 \times 10^{-7}$
	SEG39	$1.3 \times 10^{-10} \pm <1.0 \times 10^{-12}$	$7.2 \times 10^{5} \pm 8.3 \times 10^{2}$	$9.1 \times 10^{-5} \pm 4.0 \times 10^{-7}$
	SEG41	$1.9 \times 10^{-10} \pm 2.7 \times 10^{-12}$	$1.1 \times 10^{6} \pm 2.6 \times 10^{3}$	$2.1 \times 10^{-4} \pm 5.5 \times 10^{-7}$
**Recombinant lab-made SEH**	SEH1	$1.1 \times 10^{-9} \pm 3.7 \times 10^{-12}$	$1.1 \times 10^{6} \pm 3.0 \times 10^{3}$	$1.1 \times 10^{-3} \pm 2.2 \times 10^{-6}$
	SEH4	$1.0 \times 10^{-9} \pm 3.6 \times 10^{-12}$	$6.4 \times 10^{5} \pm 1.9 \times 10^{3}$	$6.4 \times 10^{-4} \pm 1.3 \times 10^{-6}$
	SEH6	$9.3 \times 10^{-11} \pm <1.0 \times 10^{-12}$	$1.9 \times 10^{6} \pm 3.0 \times 10^{3}$	$1.8 \times 10^{-4} \pm 9.7 \times 10^{-7}$
	SEH8	$1.1 \times 10^{-10} \pm <1.0 \times 10^{-12}$	$1.2 \times 10^{6} \pm 1.6 \times 10^{3}$	$1.3 \times 10^{-4} \pm 4.6 \times 10^{-7}$
	SEH11	$6.8 \times 10^{-11} \pm <1.0 \times 10^{-12}$	$1.9 \times 10^{6} \pm 3.8 \times 10^{3}$	$1.3 \times 10^{-4} \pm 1.2 \times 10^{-6}$

	SEH14	5.6×10^{-11}	±	$<1.0 \times 10^{-12}$	2.3×10^{6}	±	5.1×10^{3}	1.3×10^{-4}	±	1.3×10^{-6}
	SEH16	3.8×10^{-10}	±	1.6×10^{-12}	1.0×10^{6}	±	3.0×10^{3}	3.8×10^{-4}	±	1.2×10^{-6}
	SEH19	3.8×10^{-10}	±	1.8×10^{-12}	8.0×10^{5}	±	2.5×10^{3}	3.0×10^{-4}	±	1.1×10^{-6}
**Recombinant lab-made SEI**	SEI26	3.5×10^{-9}	±	1.7×10^{-11}	1.4×10^{5}	±	5.4×10^{2}	5.1×10^{-4}	±	1.6×10^{-6}
	SEI27	2.9×10^{-10}	±	4.1×10^{-12}	2.3×10^{5}	±	7.9×10^{2}	6.7×10^{-5}	±	9.1×10^{-7}
	SEI32	1.1×10^{-9}	±	4.8×10^{-12}	2.8×10^{5}	±	8.6×10^{2}	3.0×10^{-4}	±	9.8×10^{-7}
	SEI36	2.0×10^{-10}	±	1.1×10^{-12}	3.9×10^{5}	±	5.2×10^{2}	7.6×10^{-5}	±	4.3×10^{-7}
	SEI39	1.3×10^{-10}	±	1.3×10^{-12}	6.5×10^{5}	±	1.1×10^{3}	8.6×10^{-5}	±	8.1×10^{-7}
	SEI44	1.2×10^{-10}	±	1.0×10^{-12}	3.3×10^{5}	±	3.9×10^{2}	4.0×10^{-5}	±	3.3×10^{-7}
